# Bacteria-Templated NiO Nanoparticles/Microstructure for an Enzymeless Glucose Sensor

**DOI:** 10.3390/ijms17071104

**Published:** 2016-07-11

**Authors:** Settu Vaidyanathan, Jong-Yuh Cherng, An-Cheng Sun, Chien-Yen Chen

**Affiliations:** 1 Department of Chemistry and Biochemistry, National Chung Cheng University, 168 University Road, Min-Hsiung, Chia-Yi 62102, Taiwan; svaidynathan@gmail.com; 2 Department of Chemical Engineering & Materials Science, Yuan Ze University. No. 135 Yuandong Road, Zhongli District, Taoyuan City 320, Taiwan; acsun@saturn.yzu.edu.tw; 3 Department of Earth and Environmental Sciences, National Chung Cheng University, 168 University Road, Min-Hsiung, Chia-Yi 62102, Taiwan

**Keywords:** hollow cylinder NiO (HCNiO) nanostructure, glassy carbon electrode (GCE), non-enzymatic glucose sensor, electrochemical sensing, electrocatalysis, amperometric sensors

## Abstract

The bacterial-induced hollow cylinder NiO (HCNiO) nanomaterial was utilized for the enzymeless (without GOx) detection of glucose in basic conditions. The determination of glucose in 0.05 M NaOH solution with high sensitivity was performed using cyclic voltammetry (CV) and amperometry (i–t). The fundamental electrochemical parameters were analyzed and the obtained values of diffusion coefficient (*D*), heterogeneous rate constant (*k*_s_), electroactive surface coverage (*Г*), and transfer coefficient (*alpha-α*) are 1.75 × 10^−6^ cm^2^/s, 57.65 M^−1^·s^−1^, 1.45 × 10^−10^ mol/cm^2^, and 0.52 respectively. The peak current of the i–t method shows two dynamic linear ranges of calibration curves 0.2 to 3.5 µM and 0.5 to 250 µM for the glucose electro-oxidation. The Ni^2+^/Ni^3+^ couple with the HCNiO electrode and the electrocatalytic properties were found to be sensitive to the glucose oxidation. The green chemistry of NiO preparation from bacteria and the high catalytic ability of the oxyhydroxide (NiOOH) is the good choice for the development of a glucose sensor. The best obtained sensitivity and limit of detection (LOD) for this sensor were 3978.9 µA mM^−1^·cm^−2^ and 0.9 µM, respectively.

## 1. Introduction

Nowadays, amperometric glucose sensors are relevant for use in blood sugar monitoring with reliable sensitivity and selectivity in the health care industry. In the clinical field it was estimated that 2.8% of the world population, around 171 million people, were affected by diabetes in 2000. It will be projected to be 4.4% by the year 2030, approximately 366 million people [1]. The diagnosis of diabetes has become a far more sophisticated branch of science with increasing self-testing kits. Almost 85% of the entire biosensor market is the commercial glucose biosensor which makes diabetes a model for the development of new biosensors [2]. Biosynthesis of nanoparticles (NPs) using biological molecules and microorganisms has rapidly emerged as nanobiotechnology, i.e., biotechnology combined with nanotechnology [3]. Green nanotechnology could be a best alternative for the nanomaterial synthesis with meticulous nature with the help of variety of biological molecules, such as proteins, carbohydrates, and polyphenols [4]. NPs with different shapes, such as spherical, triangular, octahedral, cluster, and amorphous crystalline have been successfully synthesized using microorganisms. *Pseudomonas stutzeri*, *Magnetospirillum*, *Escherichia coli*, *Klebsiella aerogenes*, and *Lactobacillus* strains have been used for the bacterial shaped synthesis of silver, gold, cadmium, magnetite, and titanium NPs [5,6,7,8,9].

Most of the studies involved for the glucose sensor are the enzymatic reaction between the glucose and glucose oxidase (GOx). GOx catalyzes the oxidation of glucose to gluconolactone in the presence of redox mediators and metallic NPs coated on the electrode. However, the value of the enzyme, mediator, and electrode are expensive. In addition, electrode fouling might occur due to the thermal environment, pH change, lack of chemical stability, and fair toxicity [10,11,12]. Hence, the enzymeless glucose sensors are dramatically developed due to the low fabrication cost, overpotential reduction, and low detection limit with selectivity, sensitivity, and stability [13,14,15,16,17,18].

The noble metals and their oxides used as the redox mediators for the non-enzymatic glucose sensors have been reported [19,20,21,22,23,24,25]. Further work for the sensitive determination of glucose by simple and available materials, which have low cost and are less time consuming is required. Nanomaterials of NiO become popular for sensor devices because of its magnetic and heterogeneous electrocatalytic activities [26,27,28,29,30]. Green method is preferable for the preparation of materials in chemical industry, which is cost effective and is safe to the environment and human beings [31]. NPs of HCNiO with unique bacterial morphology were synthesized and used for glucose sensor in this study. Nanomaterials were precipitated due to the urea hydrolysis induced by bacterial metabolism [32,33]. The role of bacteria is to produce urease which catalyzes the hydrolysis of urea to CO_3_^2−^ and NH^4+^ ions. Those ions result in an increasing pH which favors carboxylate ion (−COO^−^) on the bacterial cell wall to bind with the positive nickel ion providing a nucleation site. The CO_3_^2−^ from urea hydrolysis reacts with nickel ions to form Ni precipitate on the surface of bacterial template. This is the formation of bio-Ni precipitate on the cell-wall structure.

The aim of this study is to develop the enzymeless electrode for the glucose detection at lower concentration without any additives, such as enzymes and expensive metallic NPs. The glassy carbon electrode, with the help of bacterial-shaped hollow cylinder NiO (HCNiO) nanomaterial was used as a sensor and its properties were studied. This sensor showed excellent electrocatalytic activity towards the glucose detection, and was investigated by cyclic voltammetry and amperometry i–t techniques.

## 2. Results and Discussions

### 2.1. Electrochemical Properties of the HCNiO/GC-Modified Electrode

**Scheme 1:**
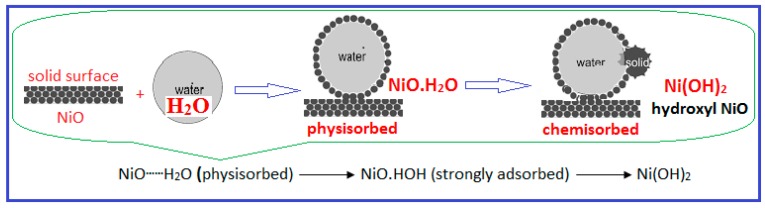
Pictorial representation of the reaction mechanism.

**Figure 1:**
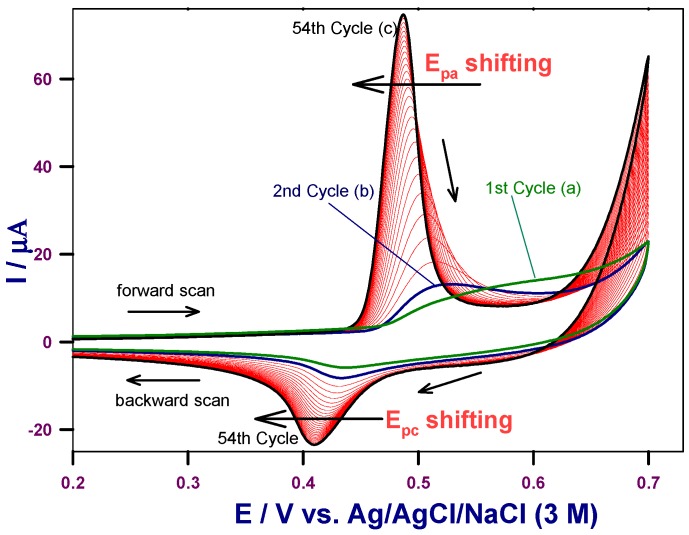
Cyclic voltammograms of the HCNiO/GC electrode (a) first cycle; (b) second cycle; and (c) 54th cycle in the potential between 0.2 V to 0.7 V in 0.05 M NaOH solution, at a scan rate of 0.05 V·s^−1^.

The electrocatalytic activity of the constructed HCNiO/GC electrode was examined in 0.05 M NaOH using CV experiments within the potential range of 0.2 V to 0.7 V. The characteristic shape of the CV curves for HCNiO/GC in an alkaline medium is shown in Figure 1. The HCNiO/GC electrode was pre-dipped in NaOH solution for a while, developed the redox peak for Ni^3+^/Ni^2+^ couple in the initial first scan but, at the same time, the sharp redox peaks were not observed for the freshly-modified electrode due to scarcity of Ni(OH)_2_. This indicated that the need of aqueous OH^−^ for the film formation of the hydrous Ni(II) oxide species and Ni(OH)_2_ spontaneously on the electrode then leads to NiOOH formation [34,35,36]. In addition, a basic medium with the suitable concentration, 0.05 M NaOH is essential for the nickel-based material for the enhancement of the catalytic activity for the sensitive oxidation of carbohydrates [37]. The anodic and cathodic peaks at a potential of 0.49 V and 0.41 V are represented by the formation of NiOOH and Ni(OH)_2_, respectively, on the solid surface of the HCNiO/GC electrode in the liquid NaOH medium. The deposition of the Ni^2+^/Ni^3+^ redox couple was confirmed by the continuous increment of the anodic and cathodic peak currents during the successive CV scan. This implied that the modified electrode in the hydroxyl group environment produced enough of the redox couple on the surface of electrode, are extremely sensitive for shuttling of electrons between the solid surface and liquid medium [38,39]. The overall scheme (Scheme 1) of the reaction mechanism (Equations (1) and (2)) for Figure 1 is suggested below:

(1) $${\text{NiO}+\text{OH}}^{-}\to {\text{NiOOH}+e}^{-}$$

(2) $${\text{Ni}\left(\text{OH}\right)}_{2}+{\text{OH}}^{-}\overset{\text{Oxidation }\left(\text{Oxd}\right)}{\underset{\text{Reduction }\left(\text{Red}\right)}{⇄}}{\text{NiOOH}+H}_{2}{O+e}^{-}$$

Thus, the couple at half-wave potential (*E_p/2_*) = 0.45 V and Δ*E_p_* = 0.08 V values exhibited a Ni^3+^/Ni^2+^ redox with a quasi-reversible process. During the successive scan the anodic peak current (*I*_pa_) beyond the +0.6 V increased successively, which may be attributed to the electro-oxidation of OH^−^ ions to O_2_ with HO^•^ radicals as intermediates. This has also been checked with the increasing OH^−^ ion concentration of supporting electrolyte for this system. It is observed that the anodic peak potential (*E*_pa_) and cathodic peak potential (*E*_pc_) shifted in the negative direction of potentials (lower), and the peak currents (*I*_pa_ and *I*_pc_) were shifting to higher values when the scan cycle number increased, which might be due to the rapid development of NiOOH by adsorptive behavior of nucleation from Ni(OH)_2_. This is the indication of active sites of Ni^2+^/Ni^3+^ produced on the surface of HCNiO/GC electrode, which is in agreement with the similar study of Wang et al. in the Ti/TiO_2_ nanotube arrays (NTAs)/Ni electrode in which the CV scans were performed in 0.1 M NaOH solution [40]. 

### 2.2. Electrochemical Surface Characterization of the HCNiO/GC Modified Electrode

#### 2.2.1. Effect of Scan Rates in the Blank Supporting Electrolyte

**Figure 2:**
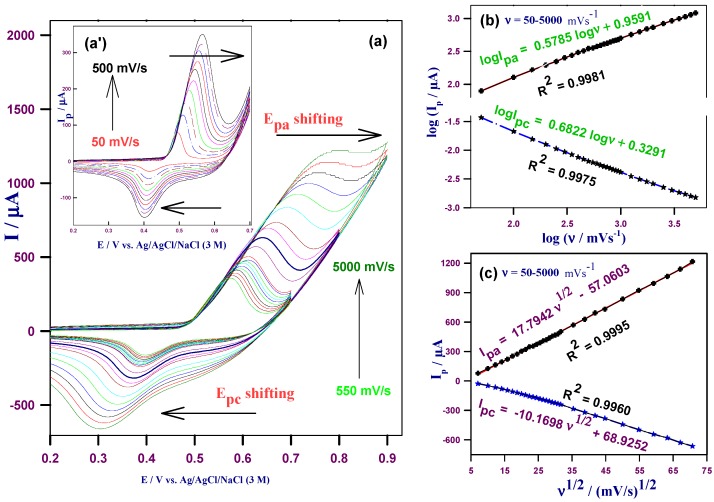
CVs of the conditioned HCNiO/GC electrode in 0.05 M NaOH at different scan rates of inset (**a’**) 0.05, 0.1, 0.15, 0.2, 0.25, 0.3, 0.35, 0.4, 0.45, 0.5 V·s^−1^ and (**a**) 0.55, 0.6, 0.65, 0.7, 0.75, 0.8, 0.85, 0.9, 0.95, 1, 1.25, 1.5, 1.75, 2, 2.5, 3, 3.5, 4, 4.5, and 5 V·s^−1^ (current response from lower to higher, respectively); (**b**) Plot of log *I*_p_ vs. log ν; and (**c**) Plot of *I*_p_ vs. ν^1/2^.

After a well production of Ni(OH)_2_/NiOOH couple by CV scanning at 0.05 V·s^−1^, the modified electrode was transferred to a fresh 0.05 M NaOH aqueous solution for further studies. Figure 2 shows the cyclic voltammograms of HCNiO/GC-modified electrode at various scan rates (ν) obtained in 0.05 M NaOH solution. The peak currents of anodic (*I*_pa_) and cathodic (*I*_pc_) are linearly proportional to the wide range of scan rates between 50 and 5000 mV·s^−1^, indicating an immobilized surface-controlled electrode process [41]. This adsorption-controlled process was confirmed by the double logarithmic (log *I*_p_ vs. log ν) plot of CV data with the slope values greater than 0.5 (0.58 and 0.68 for oxidation and reduction peaks respectively, Figure 2b). In addition, peak currents (*I*_pa_ and *I*_pc_) are linearly proportional to the square root of scan rates (ν^1/2^) at the same range of scan rates as shown in Figure 2c. Regression squared coefficient, (*R*^2^ > 0.99 for both anodic and cathodic) exists for the linearity plot of *I*_p_ vs. ν^1/2^, which indicated that the diffusion controlled process also involved. This is because the huge availability of OH^−^ ion diffusion transport from the quiescent supporting electrolyte (liquid) to the vicinity of electrode surface film (solid) and vice versa (diffusion ↔ adsorption), during the reaction shown in Equation (3), below [40].

(3) $$\overset{2+}{\text{Ni}}{\left(\text{OH}\right)}_{2(\text{solid surface})}+{\text{OH}}_{\left(\text{aqueous phase}\right)}^{-}\overset{\text{Adsorption process }(\text{Oxd})}{\underset{\text{Diffusion process }(\text{Red})}{⇄}}\overset{3+}{\text{Ni}}\;{\text{OOH}}_{\left(\text{solid surface}\right)}+e^{-}$$

However, the amount of Ni(OH)_2_ spontaneously formed at the surface due to the interaction of H_2_O or OH^−^ was found to be extremely limited since no obvious redox peaks can be observed in the initial CV scan and at the lower applied potential energy than the overpotential of the modified electrode. Further reaction processes are occurring, as shown in the Equation (2) in Section 3.1. When increasing the scan rates, the anodic peak potential (*E*_pa_) was shifted to the more positive direction and the cathodic peak (*E*_pc_) moved towards the negative potential as shown in Figure 2a and inset a’. Even though the peak current increased remarkably with increasing scan rates, but the ratio of the anodic to cathodic peak current (*I*_pa_/*I*_pc_) was above unity. It indicates that the Ni(II) to Ni(III) hydrous oxide transformation process is a quasi-reversible reaction [42,43]. This is due to the charge interaction between the positively-charged Ni, Na, and negatively-charged OH^–^ ions in the environment [44].

#### 2.2.2. Effect of Scan Rates and Electrocatalytic Effect in the Basic Glucose Solution

**Scheme 2:**
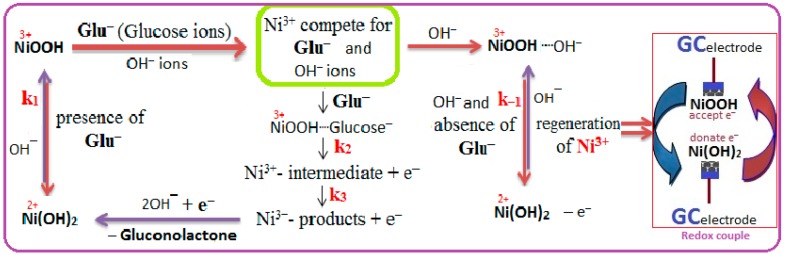
Glucose oxidation reaction mechanism under basic conditions on Ni(OH)_2_/NiOOH system.

**Figure 3:**
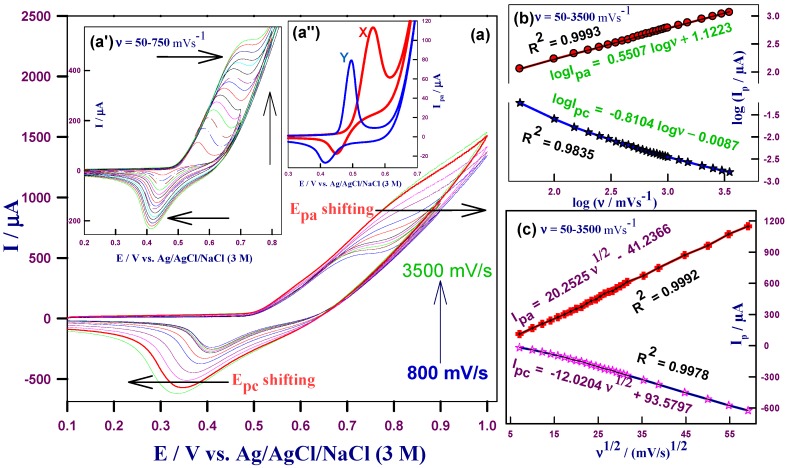
CVs of the conditioned HCNiO/GC electrode in 0.05 M NaOH containing 1 mM glucose at different scan rates of inset (**a’**) 0.05, 0.1, 0.15, 0.2, 0.25, 0.3, 0.35, 0.4, 0.45, 0.5, 0.55, 0.6, 0.65, 0.7, and 0.75 V·s^−1^ and (**a**) 0.8, 0.85, 0.9, 0.95, 1, 1.25, 1.5, 2, 2.5, 3, and 3.5 V s^−1^ (current response from lower to higher, respectively); (**b**) Plot of log *I*_p_ vs. log ν; and (**c**) plot of *I*_p_ vs. ν^1/2^. Inset (**a”**) modified electrode with 1 mM glucose (X), and without glucose (Y), in 0.05 M NaOH at the scan rate of 50 mV/s.

Figure 3 shows the effect of glucose addition on the above situation. The electroactive species of the Ni(OH)_2_/NiOOH, redox couple generated on the modified HCNiO/GC electrode was tested with (peak X in Figure 3a”) and without (peak Y in Figure 3a”) 1 mM glucose in 0.05 M NaOH solution. It can be seen that an increased *I*_pa_ from ~78 µA (blank NaOH) to ~114 µA (glucose-mixed NaOH solution) were produced at the scan rate of 50 mV·s^−1^. This excellent electrocatalytic ability is due to the rich production of NiOOH in the HCNiO material by virtue of the enhanced surface area and special morphological nature on the substrate, glassy carbon electrode (GCE) [42]. Here, the cathodic peak (*I*_pc_) decreased at higher than 50 mV·s^−1^ sweep rate but the enhancement of the glucose oxidation peak (*I*_pa_) was found (Figure 3a”) and there was no reduction peak at the lower than 15 mV·s^−1^ sweep rate. This is due to the change in the Ni^2+^/Ni^3+^ concentration ratio. The redox reaction occurring on the electrode surface (Ni^2+^↔Ni^3+^) is restricted due to the limited OH^−^ diffusion rate with the presence of glucose ions, which in turn limits the production of Ni(OH)_2_ from NiOOH, resulting from the direct electrocatalytic oxidation of glucose to gluconolactone, as showed in Equation (4). The simplest proposed reaction mechanism and its rate constant for the above processes are shown in the following Scheme 2 and Equation (4) [42,45]: 

By simply,

(4) $$\text{Ni}\left(\text{II}\right)-e^{-}\overset{\;k_{1},\text{RDS}\;}{\underset{\;k_{-1}\;}{↔}}\text{Ni}\left(\text{II}\right)\text{ and Ni}\left(\text{III}\right)+\text{Glucose}\overset{\;k_{2},k_{3}\;}{\to }\text{Ni}\left(\text{II}\right)+\text{Gluconolactone}\;k_{1}=k{}^{\circ}{\text{exp}}^{-}\left[\frac{\propto nF\eta }{\;RT}\right]\left(\text{oxd}\right)\;;\;k_{–1}=k{}^{\circ}\text{exp}\left[\frac{\left(1-\propto \right)nF\eta }{\;RT}\right]\left(\text{red}\right)$$

In the above expressions the rate constants *k*_1_ (forward-anodic) and k_−1_ (reverse-cathodic) are obviously potential-dependent for the redox reaction, k° is a standard-rate constant, η is the overpotential, and other parameters are of their usual meaning. The peak potentials (*E*_pa_) shifted towards the positive direction (higher *E*_pa_), which indicated that the diffusion limitation of glucose in the catalytic process (Figure 3a). The result might show glucose diffusion from the bulk solution phase to the surface of modified electrode in which NiOOH (Ni^3+^ active sites) formation and glucose oxidation simultaneously occurred. Adsorption of glucose is more competitive than OH^−^ on the surface of the electrode when the presence of glucose and OH^−^ ions diffused from the solution. Hence, the higher potential was required to form more Ni^3+^ sites from already-covered Ni^2+^ sites on the surface for the presence of glucose at the higher scan rates [45,46,47]. This demonstrated the certainty of electrocatalytic oxidation of glucose. In addition, there was no poisoning effect on the surface by the consistent CV run on this system. The effect of scan rates was almost similar for the absence and the presence of 1 mM glucose in 0.05 M NaOH system (Figure 2 and Figure 3). The potential sweep rates between the ranges of 50 to 3500 mV/s were used for the presence of glucose as in Figure 3. It can be seen in Figure 2a and Figure 3a, the potential shifting (both *E*_pa_ and *E*_pc_) occurs upon increasing sweep rates in both the cases. However, the oxidation peak (*E*_pa_) broadening started in the case of glucose solution (Figure 3a). Eventually, at higher than 800 mV/s scan rates, a wider broadening arose that indicated the limitation was due to the charge transfer kinetics and which is also associated with the charge propagation in the surface film. A very slow e^−^ transfer occurs in the complex situation of the film through Glu^−^, Ni^3+^, Ni^2+^, and regenerated Ni^3+^ when abrupt increasing the sweep rate. This could barrier for the diffusion of ions and migration of e^-^s. It could also involve the chemical reactions between the ions in the solution (OH^−^, Glu^−^) and the nickel active sites on the surface (i.e., EC’ mechanism). In addition, the polarizability of the ions might interfere for free movement (in and out) through the film. This similar diffusion process and the rate of the reaction have been already reported for other nickel-modified electrodes [43,44,48]. Figure 3b–c show the linear relationships for adsorption controlled and diffusion controlled processes with their regression (*R*^2^ > 0.99) and slope values greater than 0.5 (*I*_pa_ ~ 0.55 and *I*_pc_ ~ 0.81), respectively [49]. This same range of values was obtained in the case of the absence of the glucose experiment (Figure 2). This diffusion process is the rate-determining step (RDS) of the total redox process on the film when the presence of glucose, OH^−^ ions and counter Na^+^ ions (for charge neutralization of the film) [41,43,49]. The surface coverage concentration (Г) of HCNiO/GC was evaluated from the following Equation (5)

(5) $$\Gamma =\frac{Q}{\text{nFA}}$$

where A is the area of the GCE (0.071 cm^2^), n is the number of electrons involved in the redox reaction, Q is the charge obtained by integrating the anodic peak area of CV at low scan rate (*ν* = 10 mV/s) for HCNiO/GC, and F is the Faraday constant (96,500 C/mole). It is assumed that all of the immobilized redox centers are electro-active species on the voltammetry time scale. The values of surface coverage for 2 µL and 10 µL coated HCNiO/GC electrodes were 1.45 × 10^−10^ mol/cm^2^ and 1.25 × 10^−9^ mol/cm^2^, respectively, which correspond to the presence of a monolayer of effective surface species. 

#### 2.2.3. Kinetic Studies of HCNiO/GC Electrode in the Basic Glucose Solution

**Figure 4:**
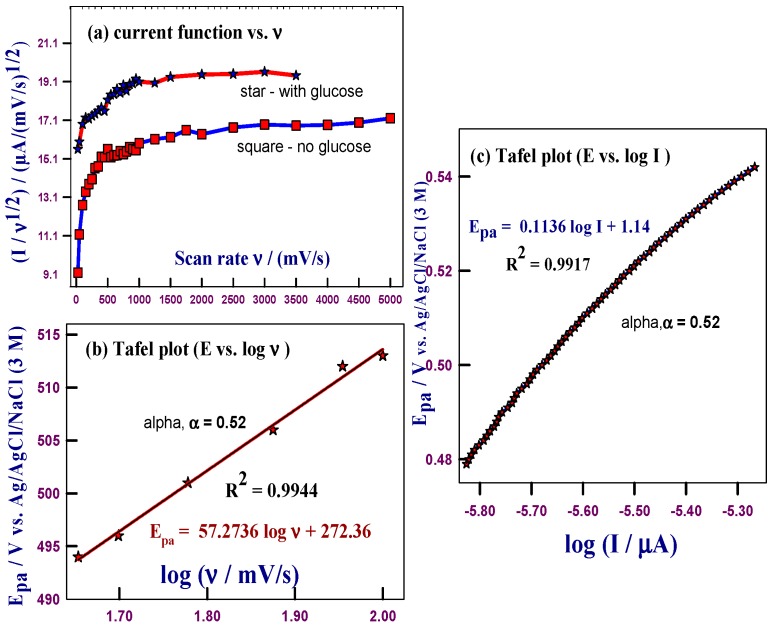
(**a**) Variations of the peak current function (I/ν^1/2^) vs. ν for the oxidation of glucose; (**b**) Dependence of the peak potential, *E*_pa_ on log ν; and (**c**) Tafel plot derived from the potentiodynamic polarization curve (*I*–*E*) at 5 mV/s for a HCNiO/GC electrode in 0.05 M NaOH in the presence of 1 mM glucose.

**Figure 5:**
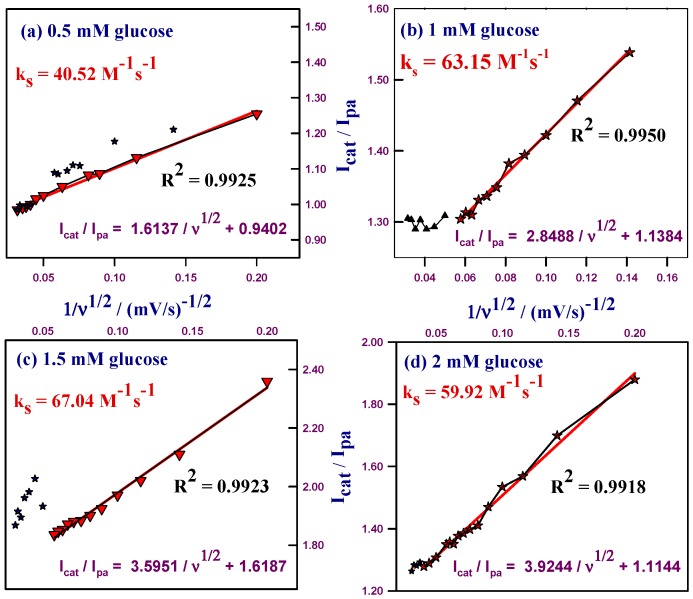
Plot of *I*_cat_/*I*_pa_ vs. 1/ν^1/2^ for rate constant studies on a HCNiO/GC electrode in different concentrations of glucose in 0.05 M NaOH at different ν (25–900 mV/s) (**a**) 0.5 mM glucose; (**b**) 1 mM glucose; (**c**) 1.5 mM glucose; and (**d**) 2 mM glucose.

Moreover, the adsorption process confirmed by the plot of the scan rate normalized current function (I/ν^1/2^) vs. scan rate (ν). Current function increased with scan rate, as shown in Figure 4a. It is expected for catalytically-coupled adsorption of an electrochemical-chemical (EC’) process. Steady linearity was found at higher ν in both the presence and absence of glucose cases, which indicates that the current function is independent of ν. This consistent fact revealed that the Ni(OH)_2_/NiOOH transition is the quasi-reversible process in the NaOH/glucose solution [44,50]. In order to obtain information on the rate determining step (RDS), the Tafel slope “b”, was determined from the Figure 4b, plot of E_p_ vs. log ν, using the following Tafel equation (Equation (6)), valid for a totally irreversible diffusion-controlled process:

(6) $$\eta =a+b\text{ log }i\;\text{or }E_{p}=\frac{b}{2}\text{ log }\nu +\text{constant}$$

where “a” and “b” are Tafel constants $\frac{2.3RT}{\alpha F}$ log i_o_ and $\frac{-2.3RT}{\alpha F}$, respectively; and i and i_o_ are current and exchange current, respectively [51]. The partial derivative of *E* vs. log ν plot is $\frac{\partial E}{\partial \text{ log }\nu }$ , equal to the Tafel slope b/2. Slope b/2 obtained for this work is 0.0573 V/decade, so, *b* = 0.1146 V/decade. This slope indicates that a one electron transfer process is the RDS (slow) with the electronic transfer coefficient (α = 0.52) for glucose solution. This one electron transfer (rate-limiting step) process was also confirmed by the another type of Tafel plot (*E*_pa_ vs. log *I*), which were drawn using the data from the rising part of the steady-state current–voltage (I–E) curve for electrocatalytic oxidation of glucose recorded after 30 s of polarization at the desired potential at a scan rate of 5 mV/s in 0.05 M NaOH. A slope of 0.114 V/decade (i.e., 8.77 (V/decade)^−1^) is obtained as in Figure 4c, indicating the one electron transfer process to be the rate-limiting step with the transfer coefficient of α = 0.52 [52]. The value of diffusion coefficient (D, cm^2^/s) were obtained by the Randles-Sevcik equation (Equation (7)) for irreversible systems using the slope of scan rates study (*I*_p_ vs. ν^1/2^) with 1 mM glucose solution in 0.05 M NaOH [53]:

*I*_p_ = (2.99 × 10^5^) α^1/2^ A C* D^1/2^ ν^1/2^ (7)

where C* is the bulk concentration of glucose in 0.05 M NaOH in terms of molar (mol/cm^3^) solution. In this study the obtained *D* value is 1.75 × 10^−6^ cm^2^/s from the slope value of Figure 3c, which is correlated with the reported literature; A is the electrode surface area (cm^2^) and ν is the scan rate (V·s^−1^). The rate constant of the heterogeneous electron transfer (*k_s_*) reaction between basic glucose solution and electro-generated Ni(II)/Ni(III) couple on the solid surface can be calculated by the CV technique [50]. Here, this system involved a second-order rate constant *k_s_* (M^−1^·s^−1^) because OH^−^ and Glu^−^ ions are necessary for the oxidation. It may be affected by specific adsorption, surface solvent layer, nature of the electrode material itself (GCE), and the film on the electrode surface. There is no bond formation and bond dissociation in this case [53,54]. The rate constant (*k_s_*) was calculated using the following Nicholson and Shain’s interpreted equation (Equation (8)) [50]:

(8) $$\frac{I_{\text{cat}}}{I_{\text{pa}}}=(\frac{1}{0.447}){\left(\frac{RT}{nF}\right)}^{1/2}{\left(\frac{k_{s}C}{\nu }\right)}^{1/2}$$

where *I*_cat_ is the catalytic oxidation peak current with known glucose concentration (C) and *I*_pa_ is the diffusion current of oxidation peak without glucose in 0.05 M NaOH. The linearity plot of (*I*_cat_/*I*_pa_) vs. (1/ν)^1/2^ arrived with the different potential scan rates of 25 to 900 mV/s CV run in the basic glucose and basic solutions separately. The rate constant values 40.52, 63.15, 67.04, and 59.92 M^−1^·s^−1^ were obtained for different glucose concentrations 0.5, 1, 1.5, and 2 mM in 0.05 M NaOH solution as shown in Figure 5a–d, respectively. When increasing the concentration of glucose from 0.5 to 1.5 mM, the rate constant also increased gradually, and then decreased, starting at 2 mM glucose, as shown in the Figure 5d. This implied that the limitation of the rate constant occurred which could be attributed to the saturation of the active sites by the passive layer of the basic glucose molecules on the surface of the electrode [55]. It resulted that there may not be sufficient active sites for the adsorption of higher concentrations of glucose. In addition, some other chemical complications, such as the glucose intermediate occurring, could be a barrier to reach the catalytic surface of the electrode for the diffusion of glucose molecules. A high concentration of OH^−^ might be required for the oxidation of a high concentration glucose, and this part of work is under progress along with this study [50,52]. The average value of the rate constant for the concentration range of 0.5 mM to 2 mM glucose solution was 57.65 M^−1^·s^−1^, which is in agreement with the reported literature [47,50].

### 2.3. Electrocatalytic Determination of Glucose by a HCNiO/GC Electrode

#### 2.3.1. Cyclic Voltammetric (CV) Method for the Detection of Glucose

**Figure 6:**
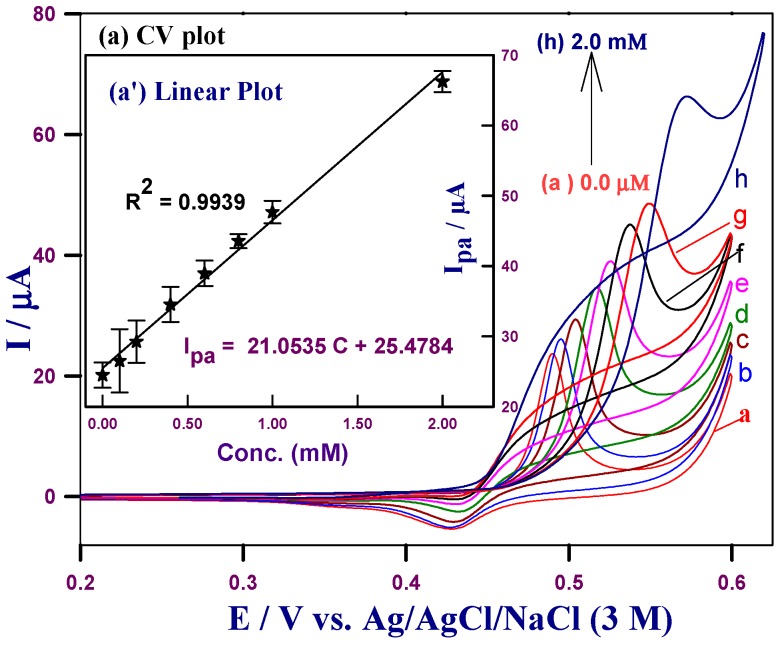
(**a**) CVs of the conditioned HCNiO/GC electrode in 0.05 M NaOH with different concentrations of glucose, (a) 0.0 mM; (b) 0.1 mM; (c) 0.2 mM; (d) 0.4 mM; (e) 0.6 mM; (f) 0.8 mM; (g) 1.0 mM; and (h) 2.0 mM at ν = 50 mV/s; and inset (**a’**) linearity plot of current vs. concentraion of the CV data. The mean peak current (*n* = 3) at the relevant concentration is shown with error bars equal to three standard deviation (3σ).

Figure 6a shows the CVs of the 2 µL HCNiO (Г = 1.45 × 10^−10^ mol/cm^2^)-coated GC electrode recorded in different glucose concentrations (0.10 mM to 2 mM) in 0.05 M NaOH solution at a 50 mV/s sweep rate. The linearity plot obtained for the current versus concentration with *R*^2^ = 0.99 from the CV data is shown in the inset (Figure 6a’). The catalytic current of a HCNiO/GC electrode increased towards the glucose oxidation and the enhancement of the anodic peak current attributed to the profound conductivity and large active surface area of the material coated on the electrode.

#### 2.3.2. Amperometric i–t Detection of Glucose

**Figure 7:**
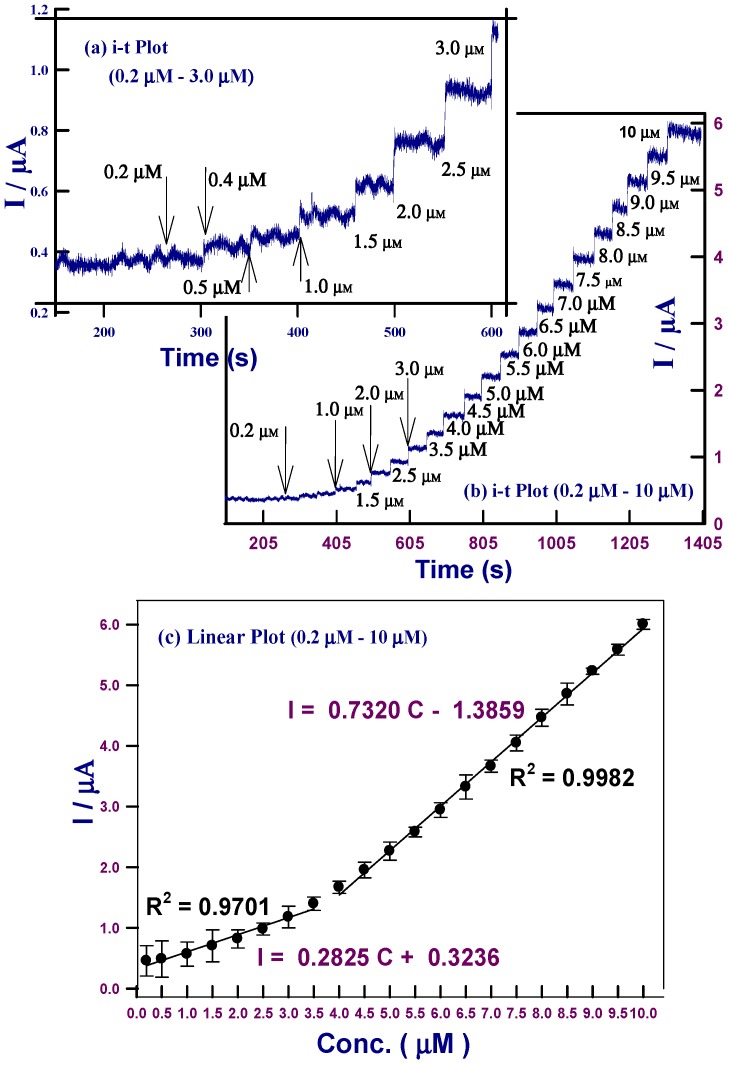
Amperometry i–t curve of a HCNiO/GC electrode in different concentrations of glucose in 0.05 M NaOH at *E* = 0.52 V; (**a**) i–t plot of 0.2 to 3 µM glucose; (**b**) i–t plot of 0.2 to 10 µM glucose; and (**c**) lnearity plot of 0.2 µM to 10 µM glucose. The mean peak current (*n* = 3) at the relevant concentration is shown with error bars equal to three standard deviation (3σ).

**Figure 8:**
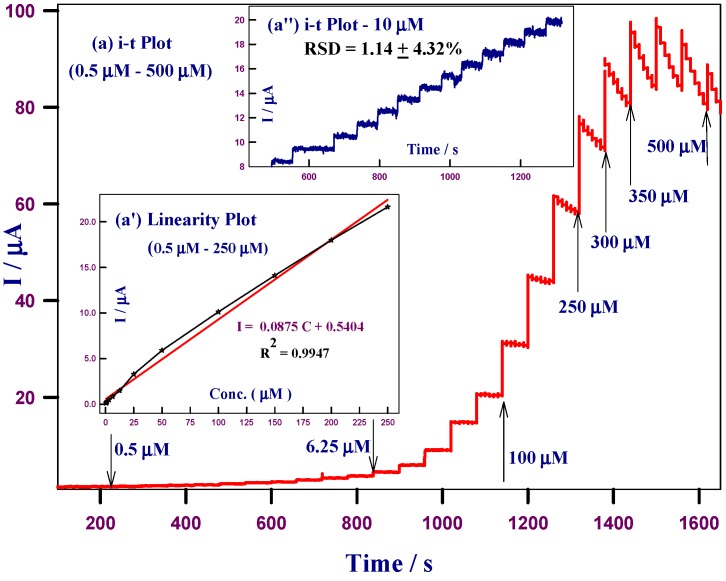
Amperometric i–t curve of a HCNiO/GC electrode in different concentrations of glucose in 0.05 M NaOH at *E* = 0.52 V; (**a**) i–t plot of 0.5 µM to 500 µM glucose; inset (**a’**) linearity plot of 0.5 µM to 250 µM glucose; and inset (**a”**) i–t plot of 10 µM glucose for Relative standard deviation (RSD).

The lower quantity of glucose oxidation can be achieved using i–t method, in which potential at 0.52 V was selected. Figure 7a,b show the amperometric sensing of glucose by successive addition of a low range of glucose concentration of 0.2 to 10 µM in a continuously-stirred 0.05 M NaOH solution. Figure 7c shows its two different calibration plot. Moreover, Figure 8a showed the current response for a higher concentration range of 0.5 to 500 µM glucose, in which the current increment was limited up to 250 µM. The current response decreased gradually after 250 µM concentration, which may be due to the diffusion dominance and adsorption difficulties of glucose ions onto the small volume of the active sites when compared to the high glucose concentration. Figure 8a’ is the linearity plot of 0.5 to 250 µM. The fast steady-state current achieved (95% of all within 3 s) and stable response upon the successive additions of 10 µM glucose with the reliable RSD value (1.14% ± 4.32%) for the 13 additions is shown in Figure 8a”. The limit of detection (LOD) was 0.9 µM measured by the standard deviation of the blank run in 0.05 M NaOH by the i–t technique (*n* = 10) and its signal was also observed by this method. The HCNiO/GC sensor exhibits much higher sensitivities of 3978.9 and 1232.4 µA mM^−1^ cm^−2^ for the linear ranges 0.2 to 3.5 µM and 0.5 to 250 µM, respectively, compared to other nickel-based GCEs reported in the literature [23]. The analytical parameters are listed in Table 1 for a comparison study. The stability and reproducibility of the sensor checked by tests every week, and a number of experiments on the same day, indicated that there was almost no change in current density for more than two weeks’ time and up to 25 tests on the same day.

**Figure 9:**
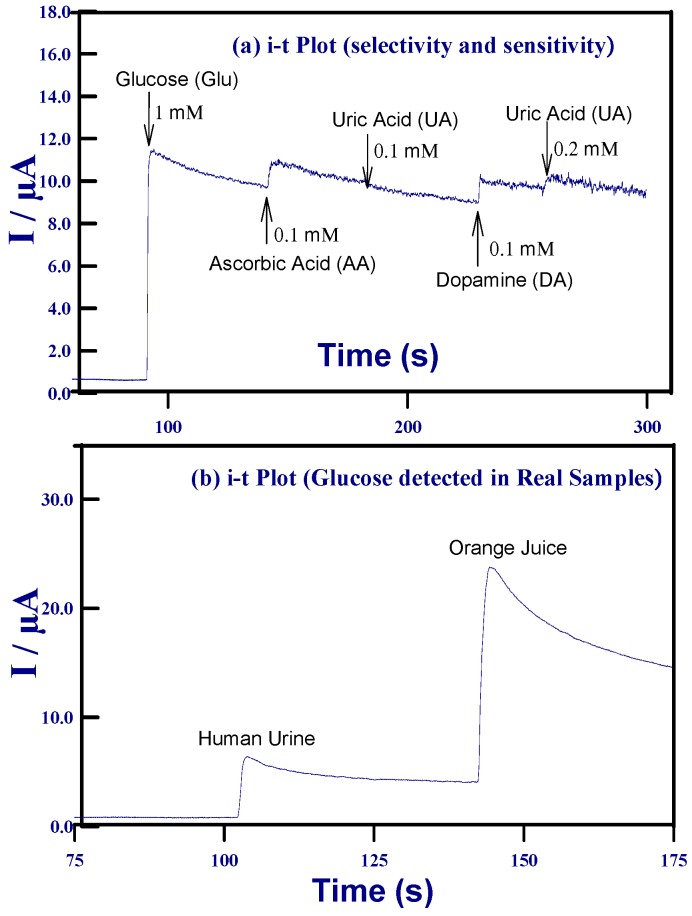
Amperometric i–t curve of a HCNiO/GC electrode in 0.05 M NaOH at *E* = 0.52 V for (**a**) an interference test in 1 mM glucose, including interferents of 0.1 mM ascorbic acid (AA), 0.1 mM uric acid (UA), 0.1 mM dopamine (DA), and 0.2 mM UA and (**b**) real samples of human urine and orange juice.

The interference test was also done to check the selectivity. The main interferents and the same oxidation potential for glucose detection in serum or other biological samples are dopamine (DA), ascorbic acid (AA), and uric acid (UA). Figure 9a expressed the selectivity by the presence of interferences of AA, UA, and DA for glucose detection. This sensor shows a better response to glucose than other interferents including AA, DA, and UA, which demonstrated good selectivity. The good result was obtained for real samples in 0.05 M NaOH, as shown in Figure 9b. This sensor could be used for the screening purposes without the invasion for the patients who suffer diabetes mellitus.

## 3. Materials and Methods

### 3.1. Reagents

NiCl_2_·6H_2_O, NaOH, urea, methanol, and ethanol were obtained from Choneye Pure Chemicals (Taipei, Taiwan), yeast from Becton Dickinson Biosciences (Taipei, Taiwan), ammonium sulfate, Tris–HCl were purchased from J.T. Baker (Chu-Bei, Taiwan), glucose from Sigma-Aldrich China Inc. (Shanghai, China), and Milli-Q (MQ-18.2 MΩ·cm at 25 °C) water were used. All chemicals were used without further purification, analytically pure, and all electrochemical experiments were carried out at ambient temperature unless otherwise stated anywhere in this paper.

### 3.2. Electrochemical Measurements

Cyclic voltammetry studies were performed on a CHI627C Electrochemical Analyzer (CH Instruments, Inc., Austin, TX, USA). The conventional three-electrode system was used throughout the electrochemical experiments consisted of a bare GCE (geometric area 0.071 cm^2^) or a modified GCE as the working electrode, a platinum wire as the auxiliary electrode, and Ag/AgCl (3 M NaCl) as the reference electrode against which all potentials were measured in this paper. For steady-state amperometric measurements, the working potential was set at 0.52 V and the solution was stirred gently with a magnetic stirrer. A digital pH meter (SUNTEX TS-1, Suntex Instruments Co., Ltd., Xinbei, Taiwan) for pH measurements and a personal computer were used for data storage and processing. 

### 3.3. Preparation of Hollow Cylinder NiO Nanostructured Material

**Figure 10:**
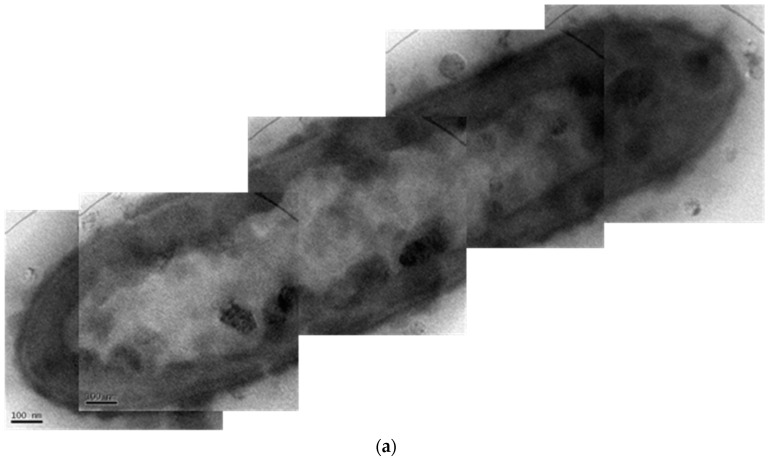

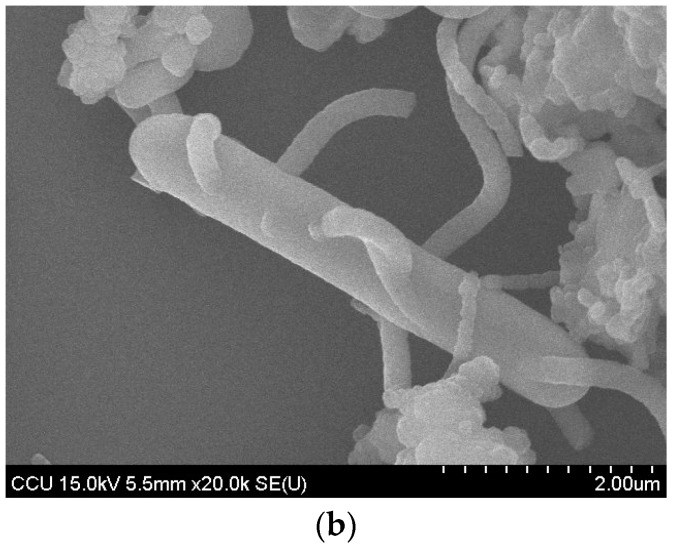
(**a**) Transmission electron microscopy (TEM) of bacterial cell wall-like HCNiO; and (**b**) Scanning electron microscope (**S**EM) of bacteria-templated HCNiO.

The synthesis procedure followed the method developed by our group [57]. Briefly, a homogeneous mixture was prepared from 0.1 M NiCl_2_·6H_2_O, 20 mL MQ water, 1.0 M urea solution, and 40 mL bacterial broth in a screw-capped Teflon tube. Bacterial culture of *Sporosarcina pasteurii* was carried out using 20 g/L yeast extract, 10 g ammonium sulfate, and 20.48 g Tris–HCl (pH of 8.5) in 1 L sterilized MQ water. The tube was then kept for one day in a mechanical shaker at 35 °C. The obtained precipitate was separated by centrifugation at 3700 rpm for 15 min. Then the precipitate was washed several times with water and, consecutively, with ethanol, dried in an air oven for 6 h at 50 °C, followed by calcination at 550 °C for 6 h. The green precipitate of bio-inorganic nickel compound changed to a black color. The hollow cylinder of microbacterial shape with nanocell wall-like structured NiO (HCNiO) compound was confirmed with the help of transmission electron microscopy (TEM) and scanning electron microscope (SEM) (see Figure 10 for TEM and SEM of bacterial shaped HCNiO). 

### 3.4. HCNiO/GC Electrode Preparation and Its Activation by CV Cycle

Prior to each experiment, the GCE was first polished with gamma alumina in water slurry using a polishing cloth and rinsed thoroughly with MQ water and ethanol. Then the required amount of HCNiO material was ultrasonically dispersed in methanol:water (20:1) solution to achieve a 10 mg/mL uniform ink. Finally, 2 µL of the ink was drop-casted onto the GCE and dried before the electrochemical experiments to study the properties of HCNiO/GCE. Then the electrode (HCNiO/GC) was conditioned between +0.2 to +0.7 V in 0.05 M NaOH, to attain the stable, well-defined peaks of Ni(OH)_2_/NiOOH film on the modified GCE by cyclic voltammetry, and the optimum 54 cycles developed as in Figure 1; its characteristic peak potentials were identified with the reported literature [10,55]. 

## 4. Conclusions

A modified GCE with bacteria-template HCNiO was successfully employed for the enzymeless detection of glucose in basic solutions. Electrochemical characteristic parameters were obtained for this well-developed redox couple on the HCNiO/GC by CV method. The detection of glucose was performed by CV and amperometric i–t techniques and LOD was found as 0.9 µM. The excellent electrocatalytic activity of HCNiO/GC sensor was stable, reproducible, and sensitive towards the detection of glucose. It is noticed that the electrode was stable for the wide range of potential scan rates up to 5 V/s with the linearity curves. Limitation of second order rate constant for the higher concentration of glucose solution was observed. The low cost of the preparation and high catalytic ability of the oxyhydroxide (NiOOH) were achieved for the electro-oxidation of glucose.

## Figures and Tables

**Table 1:** Comparison of non-enzymatic glucose sensors’ analytical parameters for different nickel-based glassy carbon electrodes (GCEs).

Serial Number	Modified GC Electrodes	Linear Range (µM)	Sensitivity	LOD (µM)	Year	Reference
1	HCNiO/GC	0.2–3.5 0.5–250	3978.9 µA·mM^−1^·cm^−2^ 1232.4 µA·mM^−1^·cm^−2^	0.9	2016	This work
2	Ni-rGO/GC	1.0–10	937 µA·mM^−1^·cm^−2^	-	2012	[23]
3	NiO/MWCNT/GC	200–12,000	-	160	2010	[55]
4	NiOOH/MWCNT/GC	250–5600	1.5 × 10^−5^ µA·M^−1^	190	2014	[53]
5	GNS/NiO/DNA-GC	1–200	14.3 µA·mM^−1^·cm^−2^	2.5	2012	[56]

GC: glassy carbon; rGO: reduced graphene oxide; MWCNT: multi-walled carbon nanotube; GNS: graphene nanosheet; DNA: deoxyribonucleic acid; LOD: limit of detection.
